# Supplementary material for: Systematic evaluation of high-throughput PBK modelling strategies for the prediction of intravenous and oral pharmacokinetics in humans
Source: Arch Toxicol. 2024 May 9;98(8):2659–76. doi: 10.1007/s00204-024-03764-9 (PMC11272695; doi:10.1007/s00204-024-03764-9)
Supplement: Supplementary file 1 — Supplementary file1 (PDF 3596 KB) [file 204_2024_3764_MOESM1_ESM.pdf]

Supplementary Information

SI-1: Extensive Description of the PK Data Retrieval Process

We downloaded all concentration-time data from the PK-DB, CvT-DB and OSP Observed Data Repository databases and filtered them for healthy, adult human subjects. We excluded all studies performed on special populations including paediatric (< 17 years), geriatric (> 60 years) or diseased populations, which we expected might display altered pharmacokinetics. Some selected conditions, like “migraine”, “psychiatric disorder”, “asthma”, “diabetes” and even “liver disease (minimal)” were permitted, however. But, for example, “liver disease (severe)” or “renal disease (end-stage)” were excluded. We filtered for profiles of administered parent compounds, discarding data of metabolites, and only selected those data that were measured in blood, plasma or serum, and only after single dose intravenous or oral administration. Further, we excluded all studies with concomitant administrations of other drugs to avoid data from drug-drug interaction studies, however, “control” or “placebo” groups from such studies were permitted. Also, we excluded endogenous compounds like ethanol or glucose. If information about both nominal dose and salt-free active pharmaceutical ingredient (API) was provided, we retrieved the salt-free API value as the dose value. When only a nominal dose was provided, we assumed this was referring to the salt-free API dose. Labels of formulations for oral administration (“tablet”, “capsule”, “solution” etc.) were extracted as provided.

After retrieving concentration-time profiles from above mentioned databases, we enriched our dataset by manually digitising more concentration-time data from the literature. For this, we followed the same strategy as before, aiming for data from healthy adults. However, in some cases, for example for certain chemotherapeutics, it proved difficult to find PK data for healthy adults or subjects younger than 60 years. Since we were able to inspect those studies manually, and to ensure that they were not performed on those special populations due to a suspected difference in pharmacokinetics, we occasionally decided to include them, if no other data on such compounds was available.

Next, we excluded data points which had a concentration value numerically close to zero (< 1e-7) after 4 h, since some studies possessed unreasonably low concentration values at the end of their concentration-time profiles, presumably representing the lower limit of quantification, or due to digitisation inaccuracies. Also, we excluded all concentration-time profiles with less than two time points. If we had retrieved more than 30 concentration-time profiles for a given compound and a given route of administration, then we excluded all profiles from individuals (where number of subjects = 1), as long as there were at least 20 other concentration-time profiles left. If the number of profiles was then still higher than 30, we excluded the studies with the least number of subjects until we had no more than 60 concentration-time profiles per study and route. Finally, we generated dose normalised plots for all compounds where multiple concentration-time curves were available to visually confirm that studies were consistent with each other and that there were no extreme outliers among the data. When we found noticeable differences between concentration-time profiles in the dose normalised plots, we looked up the corresponding original publications and either corrected or excluded those entries.

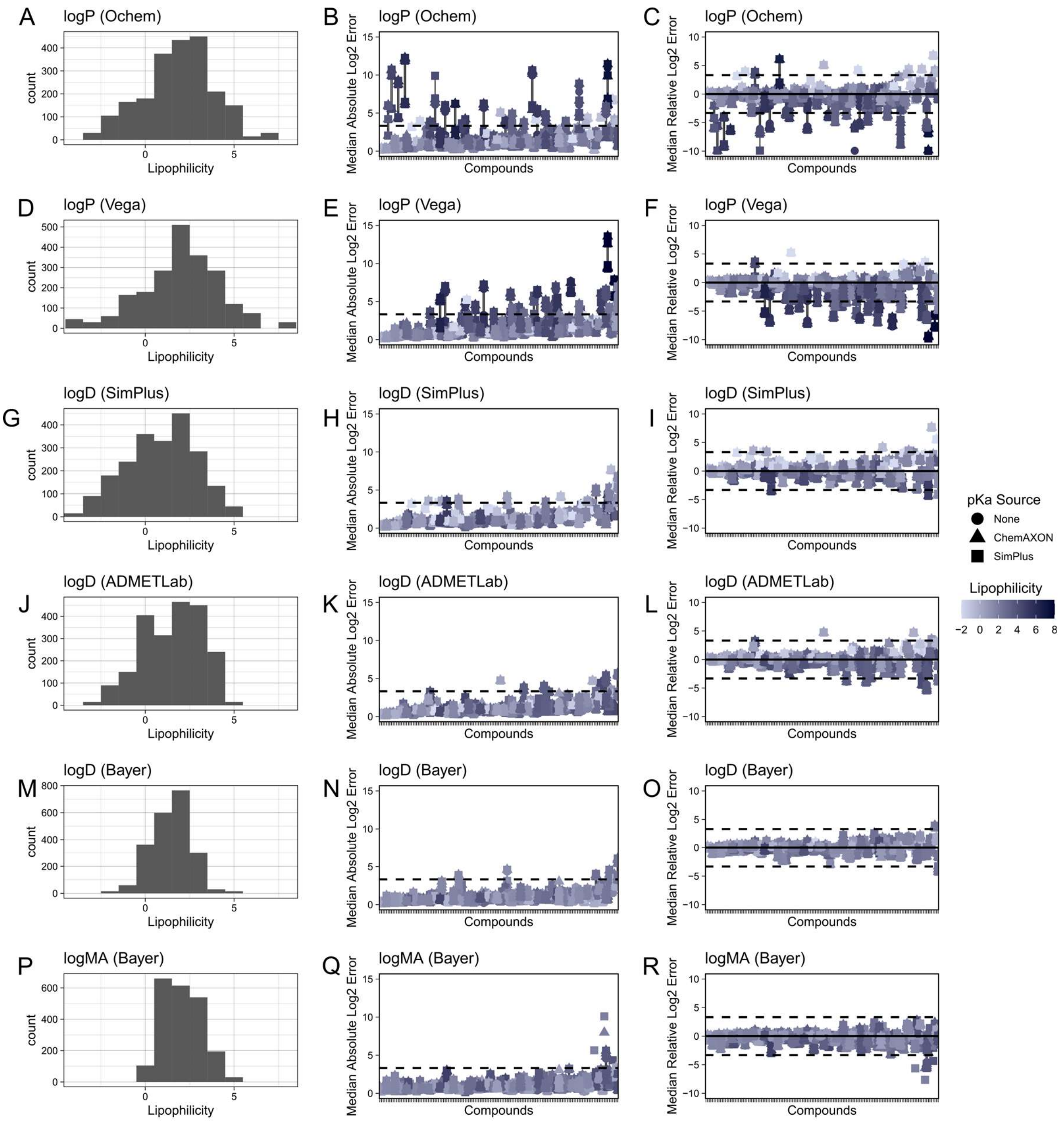

SI-Fig. 1: Comparison of predictive performances of different lipophilicity prediction tools coloured by lipophilicity value (step 1). Combinations of all available PBK model parameterisation sources were evaluated against the collected IV dataset. Clearance and fraction unbound were parameterised using *in vivo* and *in vitro* reference benchmark values, respectively. Left column shows histograms of predicted lipophilicity values by the various prediction tools. Middle column shows Median Absolute Log2 Errors of every compound, colour-coded by lipophilicity value predicted by the tool, and right column shows Median Relative Log2 Errors. Dashed lines indicate 10-fold errors.

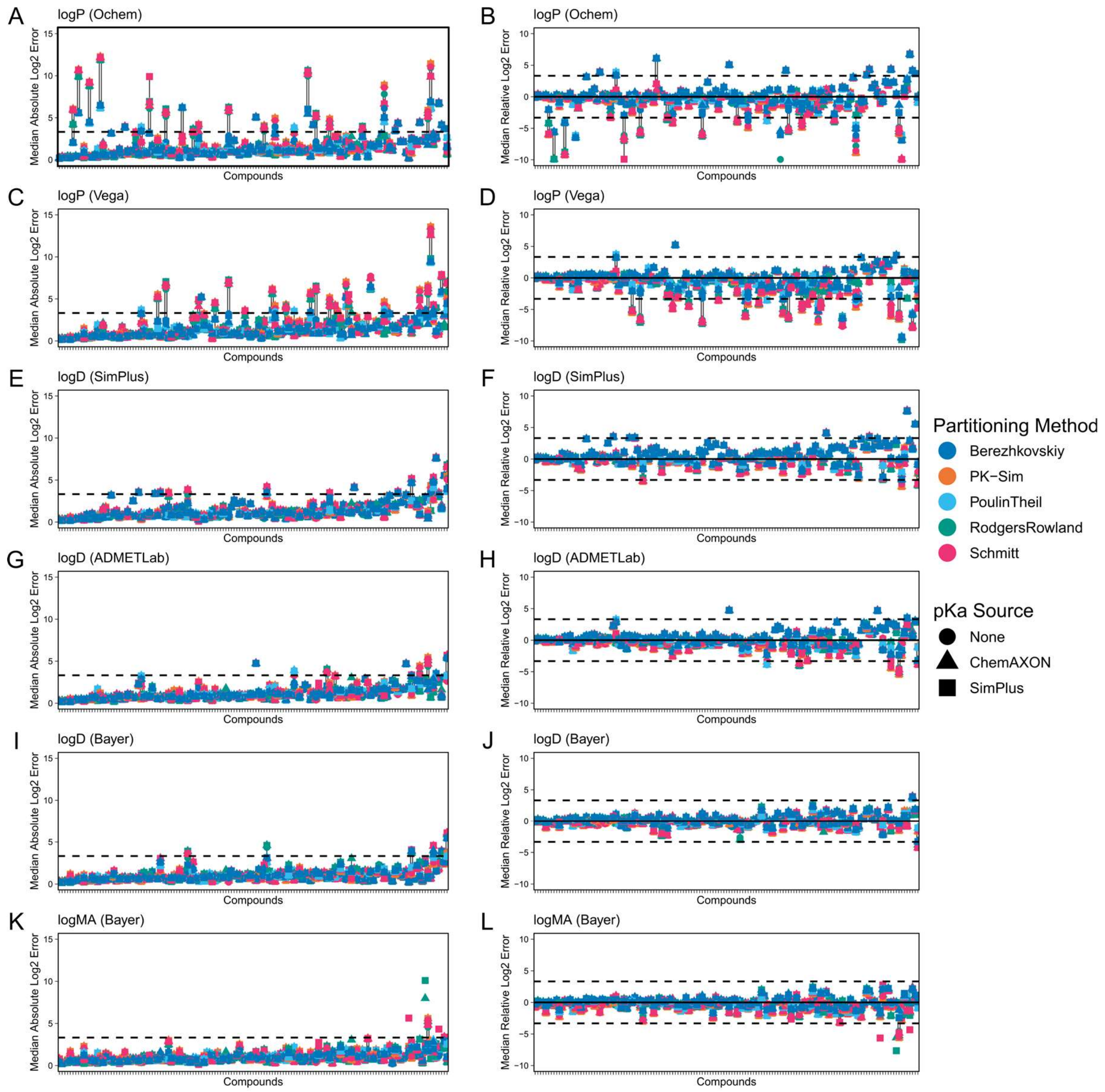

SI-Fig. 2: Comparison of predictive performances of different lipophilicity prediction tools coloured by partitioning method (step 1). Combinations of all available PBK model parameterisation sources were evaluated against the collected IV dataset. Clearance and fraction unbound were parameterised using *in vivo* and *in vitro* reference benchmark values, respectively. Left column Median Absolute Log2 Errors of every compound, colour coded by partitioning method used for simulation, and right column shows Median Relative Log2 Errors. Dashed lines indicate 10-fold errors.

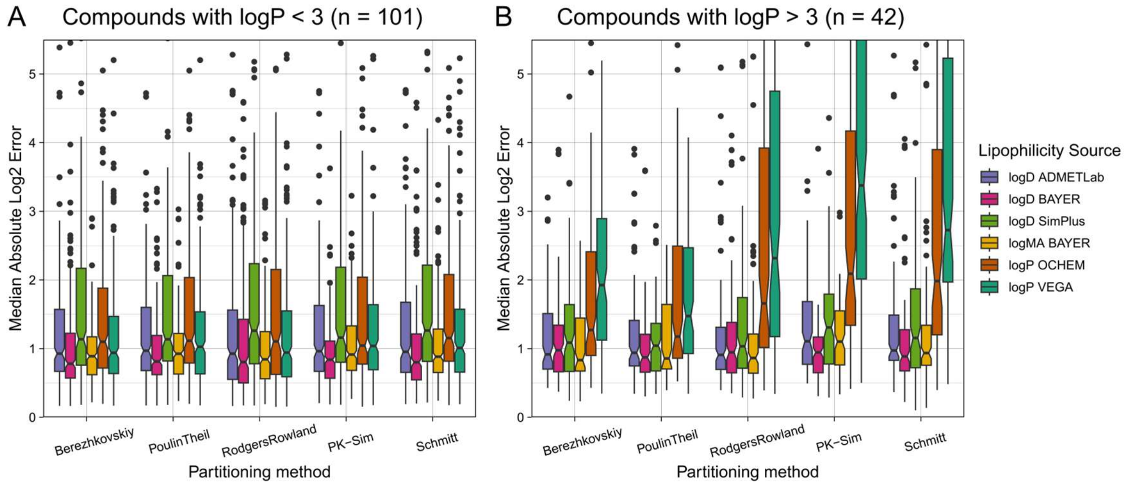

SI-Fig. 3: Comparison of predictive performances of lipophilicity prediction tools and partitioning methods separated by compound lipophilicity. Combinations of all available PBK model parameterisation sources were evaluated against the collected IV dataset. Clearance and fraction unbound were parameterised using *in vivo* and *in vitro* reference benchmark values, respectively. Lipophilicity values were set using the mentioned LogP and LogD *in silico* prediction tools. Plots show Median Absolute Log2 Errors as a measure of prediction precision. A shows results of compounds with LogP < 3 (n = 101), B shows results of compounds with LogP > 3 (n = 42).

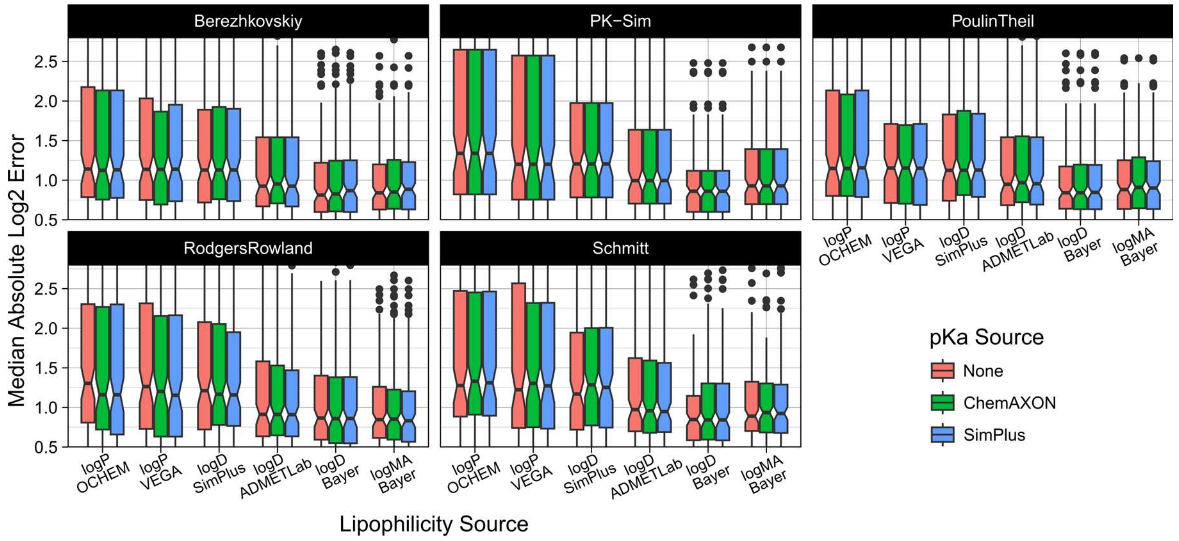

SI-Fig. 4: Comparison of predictive performances of pKa prediction tools. Combinations of all available PBK model parameterisation sources were evaluated against the collected IV dataset. Clearance and fraction unbound were parameterised using *in vivo* and *in vitro* reference benchmark values, respectively. Lipophilicity values were set using the mentioned LogP and LogD *in silico* prediction tools. Plots show Median Absolute Log2 Errors as a measure of prediction precision.

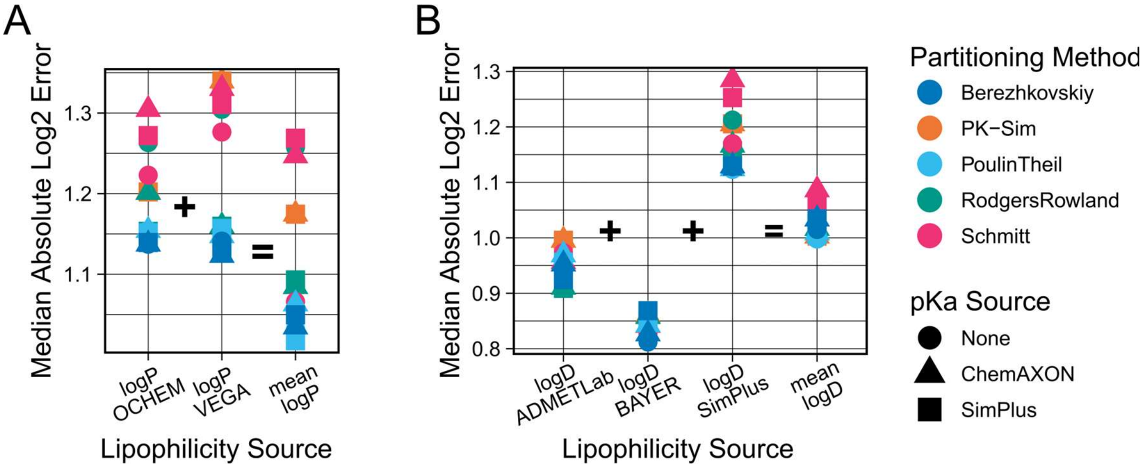

SI-Fig. 5: Predictive performances of consensus strategies for averaging lipophilicity predictions. Combinations of all available PBK model parameterisation sources were evaluated against the collected IV dataset. Clearance and fraction unbound were parameterised using *in vivo* and *in vitro* reference benchmark values, respectively. Lipophilicity values were set using the mentioned LogP and LogD *in silico* prediction tools, as well as the averages of those. Plus signs indicate which predictions of the individual LogP or LogD tools were combined to achieve the mean results marked by equal signs.

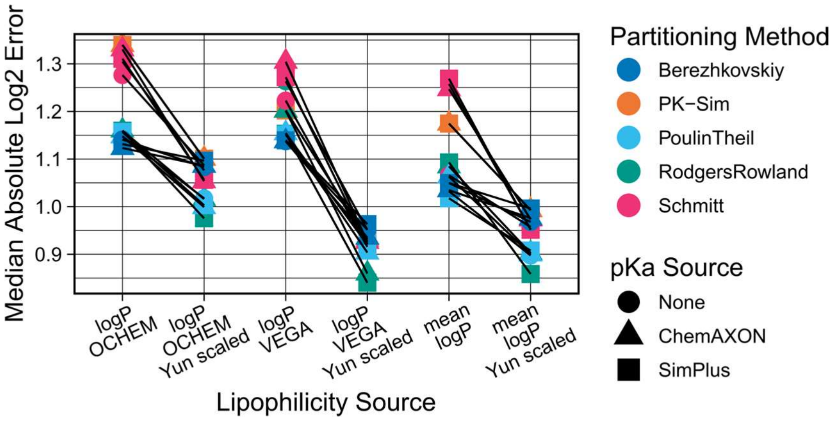

SI-Fig. 6: Change in predictive performance when using an empirical regression equation to convert LogP lipophilicity to LogMA. Combinations of all available PBK model parameterisation sources were evaluated against the collected IV dataset. Clearance and fraction unbound were parameterised using *in vivo* and *in vitro* reference benchmark values, respectively. Lipophilicity values were set using the mentioned LogP *in silico* prediction tools VEGA, OCHEM and their average. Additionally, those LogP values were converted to LogMA values by an empirical regression equation established by Yun et al. (2014).

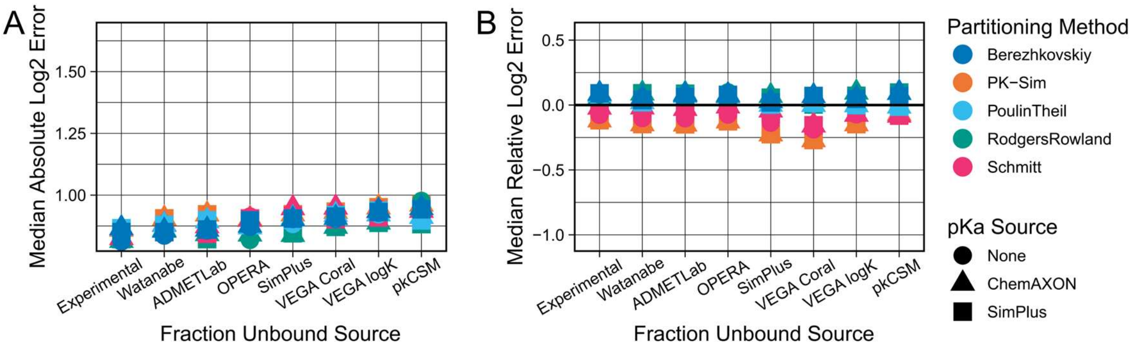

SI-Fig. 7: Comparison of Fu prediction tools when using benchmark *in vivo* plasma clearance values for PBK model parameterisation. Combinations of all available parameterisation sources were evaluated against the collected IV dataset. Results shown were generated using *in vivo* observed plasma clearance benchmark values, and the mean of the two previously determined best lipophilicity prediction tools (LogD and LogMA Bayer) as lipophilicity values. A: Median Absolute Log2 Errors as a measure of prediction precision. B: Median Relative Log2 Errors as a measure of systematic prediction bias.

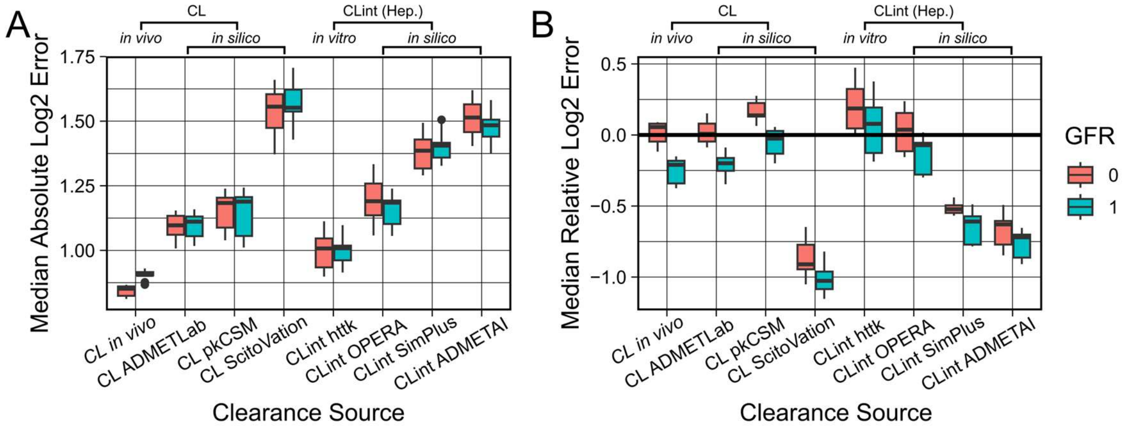

SI-Fig. 8: Effects of adding passive renal clearance on the predictive performances of HT-PBK predictions. Combinations of all available parameterisation sources were evaluated against the collected IV dataset. Results shown were generated using *in vitro* measured Fu benchmark values, and the mean of the two previously determined best lipophilicity prediction tools (LogD and LogMA Bayer) as lipophilicity values. GFR rate of 1 signifies adding passive renal clearance to the PBK model, and a value of 0 indicates leaving it out. CL refers to plasma clearance values (measured or predicted), CLint stands for hepatic intrinsic clearance (measured or predicted). A: Median Absolute Log2 Errors as a measure of prediction precision. B: Median Relative Log2 Errors as a measure of systematic prediction bias.

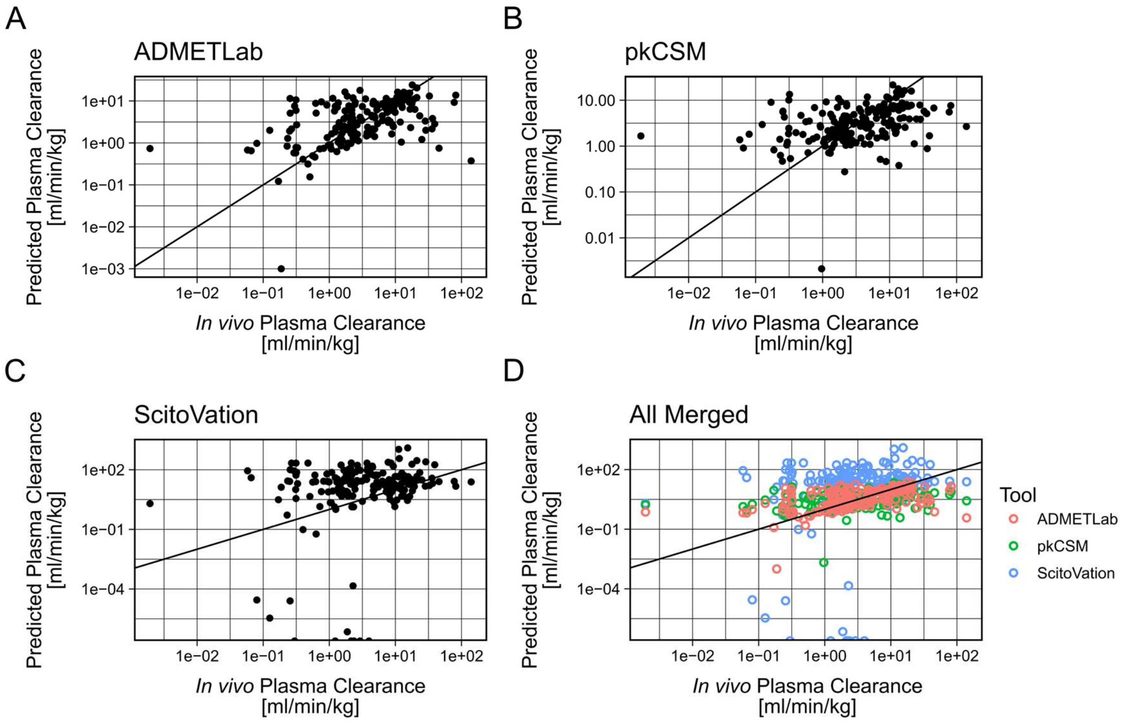

SI-Fig. 9: *In silico* predicted plasma clearance values from different tools against *in vivo* observed values. *In vivo* values were collected from the literature, *in silico* values were predicted using the corresponding tools. A-C: *In silico* predicted plasma clearance values from ADMETLab, pKCSM, and ScitoVation, respectively. D: All predicted values combined in a single plot.

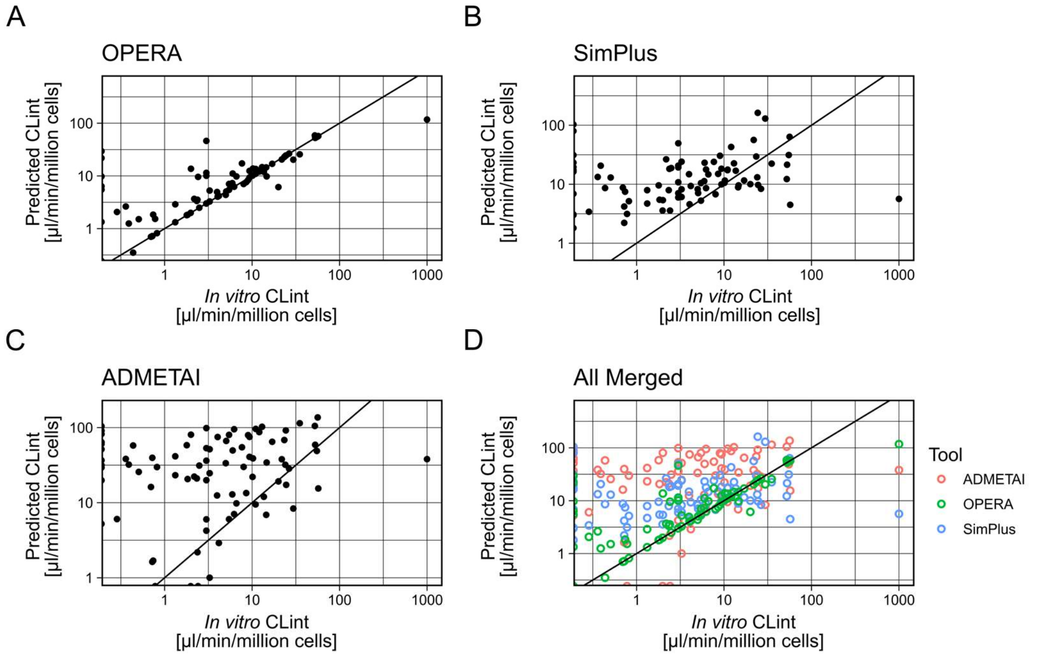

SI-Fig. 10: *In silico* predicted hepatocyte CLint values from different tools against *in vitro* measured values. *In vitro* CLint values were taken from httk version 2.2.1, *in silico* values were predicted using the corresponding tools. A-C: *In silico* predicted plasma clearance values from OPERA, SimPlus (ADMET Predictor), and ADMETAI, respectively. D: All predicted values combined in a single plot.

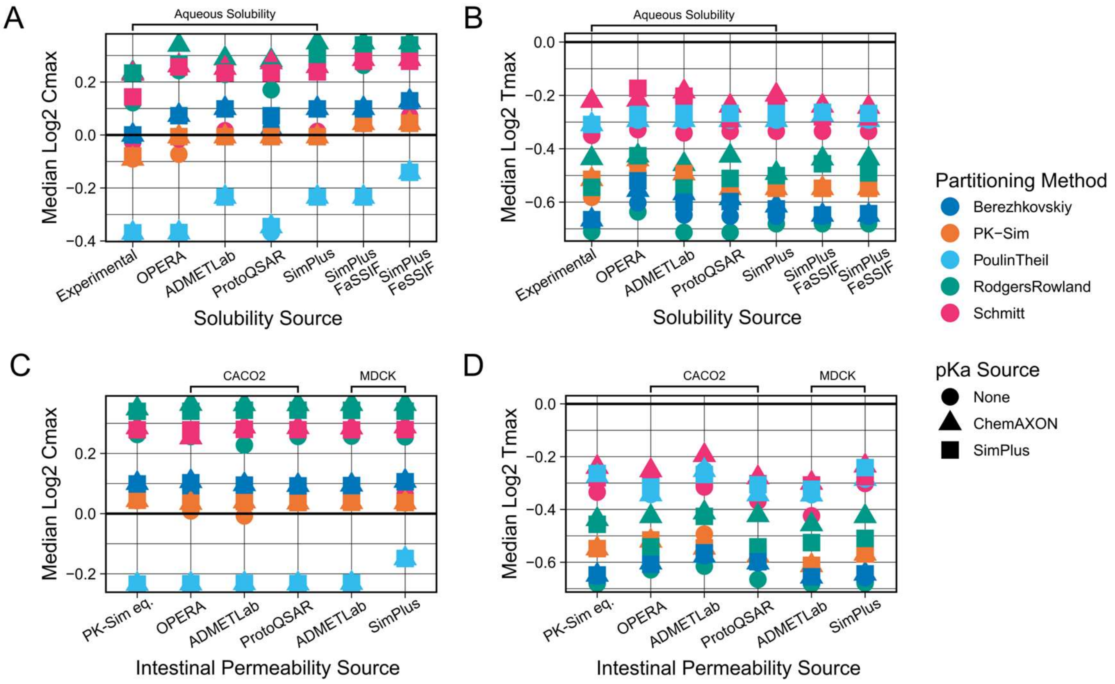

SI-Fig. 11: Cmax and Tmax prediction biases of different solubility and intestinal permeability prediction sources (step 3). Combinations of all available parameterisation sources were evaluated against the collected PO dataset (dissolved formulations). Results shown were generated using benchmark values for parameterisation of other parameters, i.e., *in vivo* plasma clearances, *in vitro* fraction unbound values and the mean of the two previously determined best lipophilicity prediction tools (LogD and LogMA Bayer) as lipophilicity values. The top row shows the Median Log2 Cmax predicted/observed (A) and the Median Log2 Tmax predicted/observed (B) for different solubility prediction methods. The bottom row shows the Median Log2 Cmax predicted/observed (C) and the Median Log2 Tmax predicted/observed (D) for different intestinal permeability prediction methods. For comparison of solubility sources, the intestinal permeability source used was the PK-Sim internal equation (PK-Sim eq.). For comparison of intestinal permeability sources, the solubility values used were the SimPlus FaSSIF values. Results for the PK-Sim internal equation were generated by inputting the Bayer LogMA predictions. Intestinal permeability predictions are either CACO2 or MDCK permeability predictions.

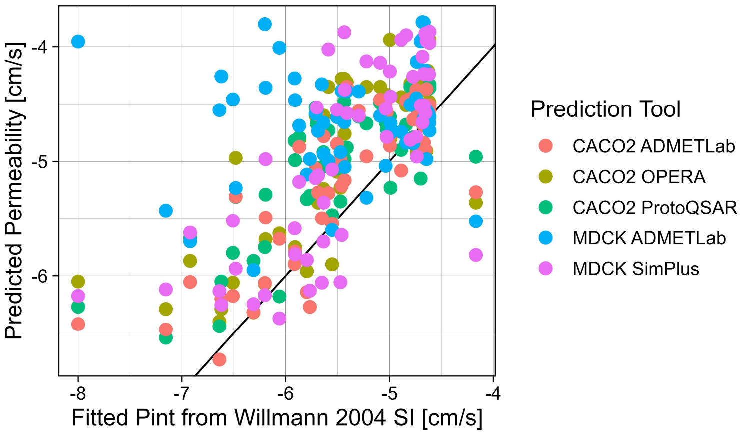

SI-Fig. 12: *In silico* predicted intestinal permeability values from different tools against optimal fitted Pint values. Optimal fitted Pint values were taken from Willmann et al. (2004).

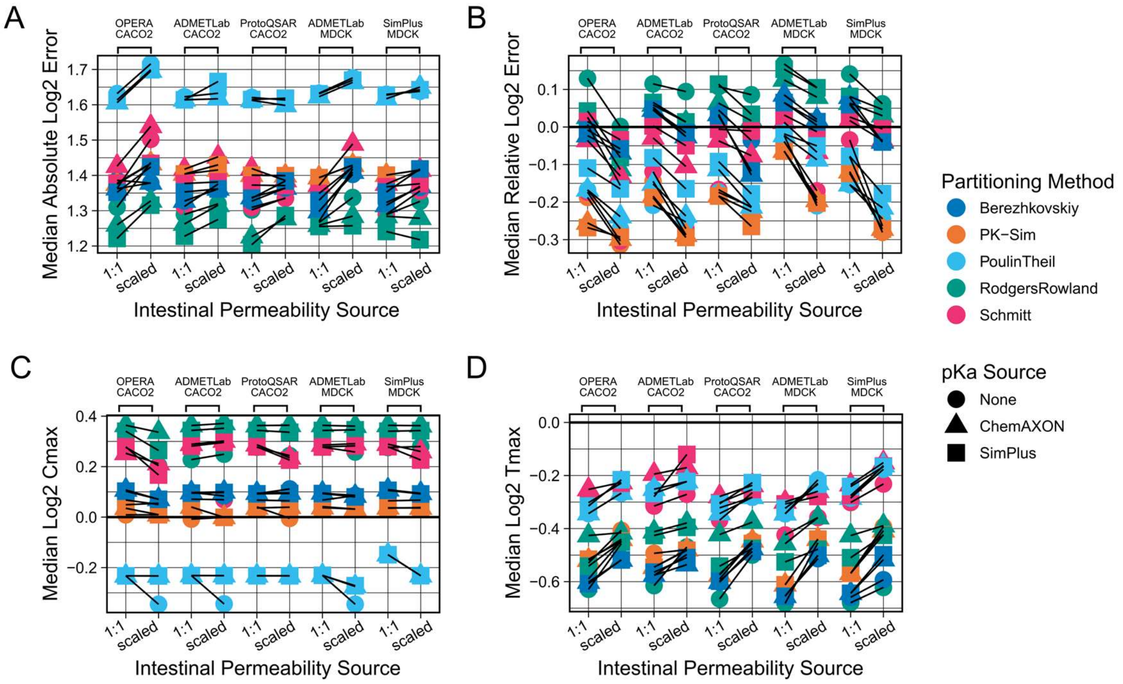

SI-Fig. 13: Effect of scaling intestinal permeability predictions on different performance metrics. Combinations of all available parameterisation sources were evaluated against the collected PO dataset (dissolved formulations). Results shown were generated using benchmark values for parameterisation of other parameters, i.e., *in vivo* plasma clearances, *in vitro* fraction unbound values and the mean of the two previously determined best lipophilicity prediction tools (LogD and LogMA Bayer) as lipophilicity values. SimPlus FaSSIF values were used as solubility values. The top row shows the Median Absolute Log2 Error (A) and the Median Relative Log2 Error (B) for different intestinal permeability prediction approaches. The bottom row shows the Median Log2 Cmax predicted/observed (C) and the Median Log2 Tmax predicted/observed (D). Lines connect the simulation results based on the same *in silico* prediction tool, either used directly (1:1) or after scaling using optimal intestinal permeability values from Willmann et al. (2004).

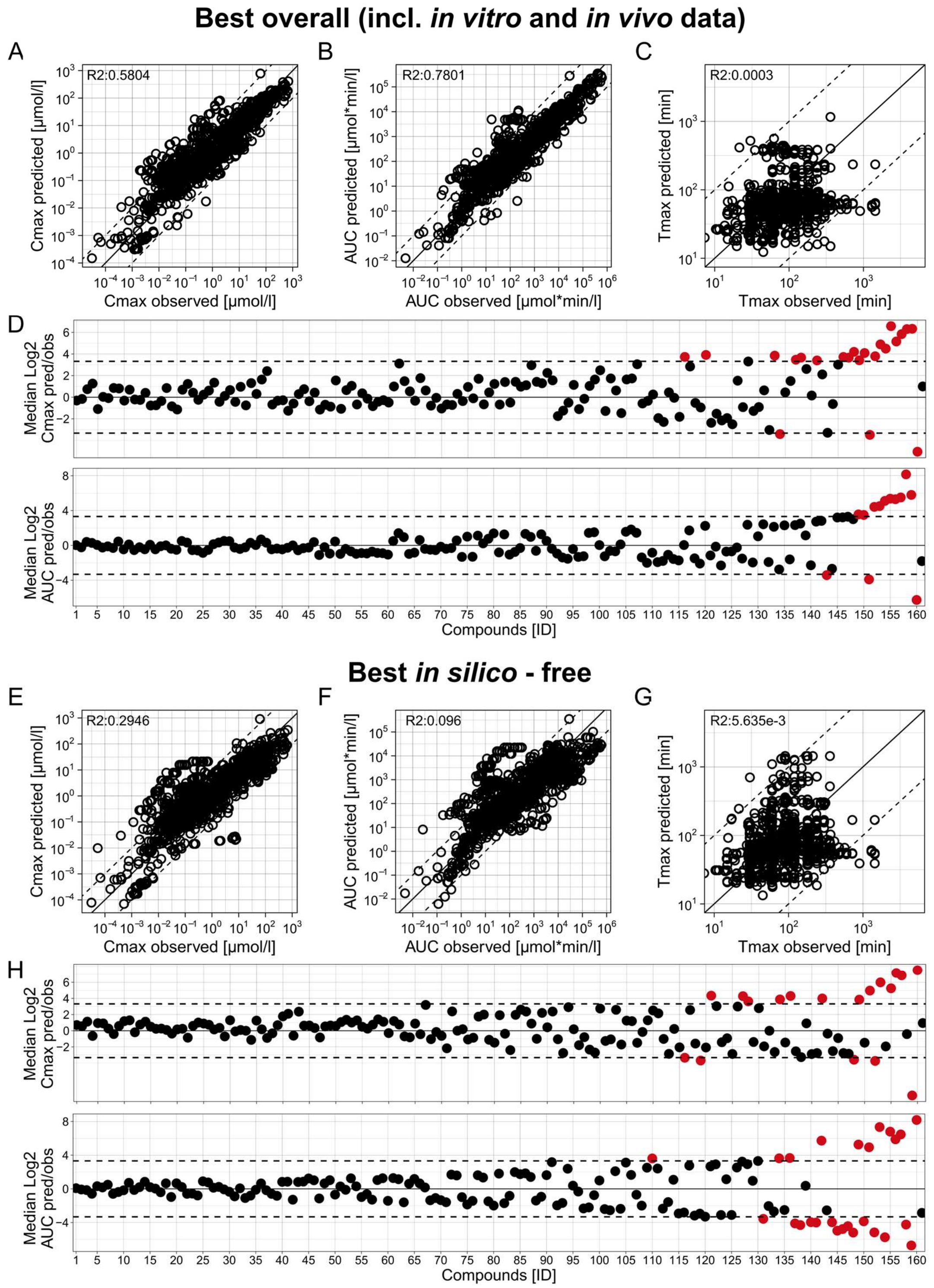

SI-Fig. 14: Predictive performances of the best overall and the best free *in silico*-based HT-PBK modelling strategy. HT-PBK models were generated following the approach outlined in Table 1, then model simulations were compared against the collected PO dataset of 161 compounds (dissolved and solid formulations). A-D: Results for the best overall strategy including *in vitro* and *in vivo* input data as reference. E-H: Results for the best HT-PBK strategy fully based on free *in silico* tools. (A+E) Predicted against observed Cmax values of all concentration-time profiles in the PO dataset. (B+F) Predicted against observed AUC values. (C+G) Predicted against observed Tmax values. (D+G) The Median Log2 Cmax and AUC predicted/observed values of each compound in the PO dataset. Dashed lines indicate 10-fold errors.

Supplementary Information References

Willmann S, Schmitt W, Keldenich J, Lippert J, Dressman JB (2004) A physiological model for the estimation of the fraction dose absorbed in humans. Journal of medicinal chemistry 47(16):4022–4031. doi: 10.1021/jm030999b

Yun YE, Cotton CA, Edginton AN (2014) Development of a decision tree to classify the most accurate tissue-specific tissue to plasma partition coefficient algorithm for a given compound. Journal of pharmacokinetics and pharmacodynamics 41(1):1–14. doi: 10.1007/s10928-013-9342-0
